# Supplementary material for: Tead1a Initiates Transcriptional Priming Through the TEAD1a/YAP‐Notch1‐Spi1/Cebpα Axis to Promote Neutrophil Fate
Source: Adv Sci (Weinh). 2025 Aug 28;12(41):e05441. doi: 10.1002/advs.202505441 (PMC12591127; doi:10.1002/advs.202505441)
Supplement: Supplementary file 4 — Supplementary Dataset [file ADVS-12-e05441-s004.zip › 3-7.zip!/3-7/02-7.cluster/cluster19/Enrich/ego_all.pdf]

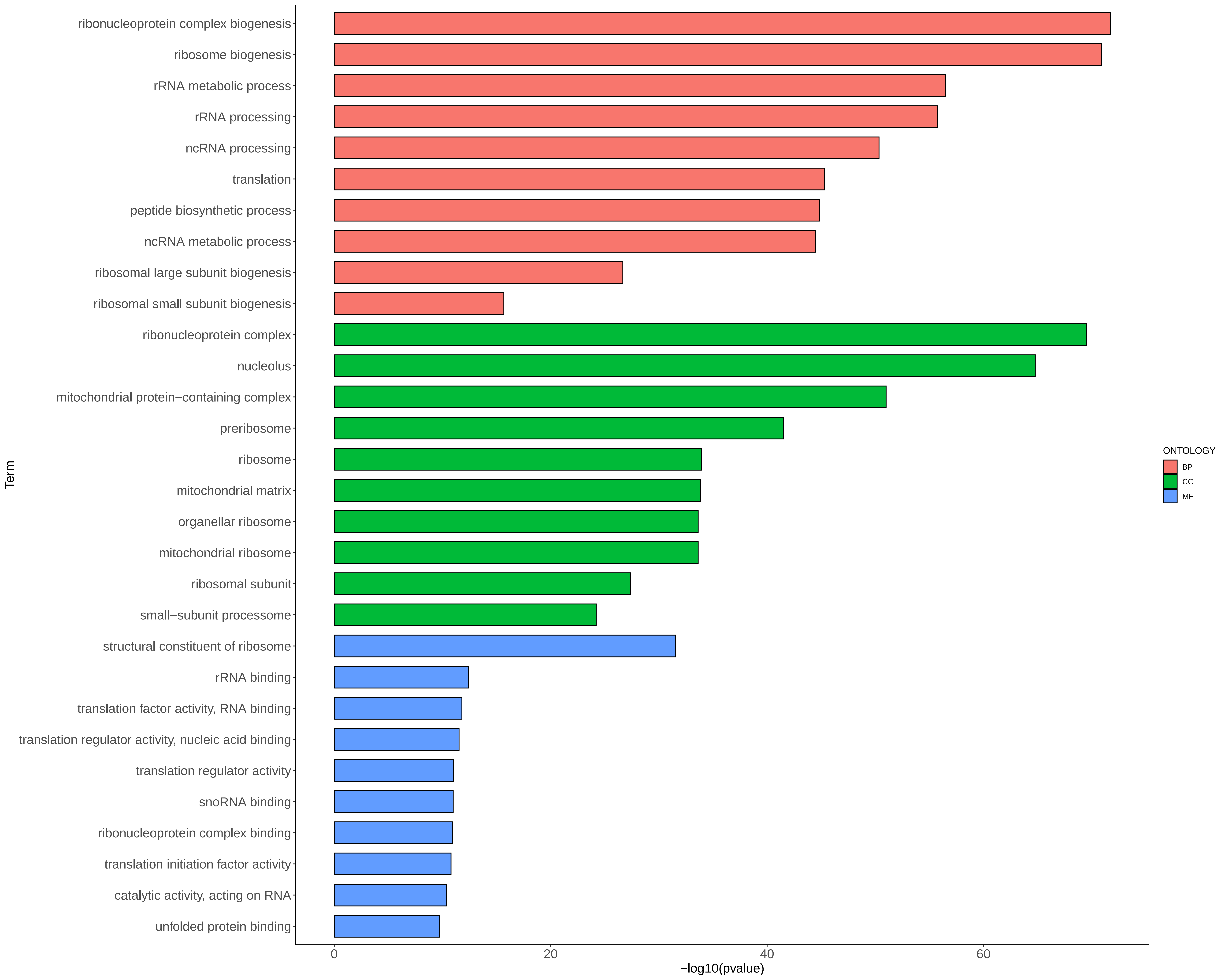

ribonucleoprotein complex biogenesis
ribosome biogenesis
rRNA metabolic process
rRNA processing
ncRNA processing
translation
peptide biosynthetic process
ncRNA metabolic process
ribosomal large subunit biogenesis
ribosomal small subunit biogenesis
ribonucleoprotein complex
nucleolus
mitochondrial protein−containing complex
preribosome
ribosome
mitochondrial matrix
organellar ribosome
mitochondrial ribosome
ribosomal subunit
small−subunit processome
structural constituent of ribosome
rRNA binding
translation factor activity, RNA binding
translation regulator activity, nucleic acid binding
translation regulator activity
snoRNA binding
ribonucleoprotein complex binding
translation initiation factor activity
catalytic activity, acting on RNA
unfolded protein binding
Term
0
20
40
60
−log10(pvalue)
ONTOLOGY
BP
CC
MF
